# Improving nutrition through biofortification–A systematic review

**DOI:** 10.3389/fnut.2022.1043655

**Published:** 2022-12-09

**Authors:** Kelvin F. Ofori, Sophia Antoniello, Marcia M. English, Alberta N. A. Aryee

**Affiliations:** ^1^Department of Human Ecology, Delaware State University, Dover, DE, United States; ^2^Department Human Nutrition, Saint Francis Xavier University, Antigonish, NS, Canada

**Keywords:** malnutrition, hidden hunger, fortification, biofortification, transgenic, agronomic, breeding, omics technology

## Abstract

Nutritious foods are essential for human health and development. However, malnutrition and hidden hunger continue to be a challenge globally. In most developing countries, access to adequate and nutritious food continues to be a challenge. Although hidden hunger is less prevalent in developed countries compared to developing countries where iron (Fe) and zinc (Zn) deficiencies are common. The United Nations (UN) 2nd Sustainable Development Goal was set to eradicate malnutrition and hidden hunger. Hidden hunger has led to numerous cases of infant and maternal mortalities, and has greatly impacted growth, development, cognitive ability, and physical working capacity. This has influenced several countries to develop interventions that could help combat malnutrition and hidden hunger. Interventions such as dietary diversification and food supplementation are being adopted. However, fortification but mainly biofortification has been projected to be the most sustainable solution to malnutrition and hidden hunger. Plant-based foods (PBFs) form a greater proportion of diets in certain populations; hence, fortification of PBFs is relevant in combating malnutrition and hidden hunger. Agronomic biofortification, plant breeding, and transgenic approaches are some currently used strategies in food crops. Crops such as cereals, legumes, oilseeds, vegetables, and fruits have been biofortified through all these three strategies. The transgenic approach is sustainable, efficient, and rapid, making it suitable for biofortification programs. Omics technology has also been introduced to improve the efficiency of the transgenic approach.

## Introduction

Food and nutrients are required in their right proportions by humans to ensure proper growth and development (1). The United Nations (UN) 2nd Sustainable Development Goal was set to eradicate extreme hunger and malnutrition whilst promoting food security (2–4). However, population growth coupled with global climate change has led to extreme hunger in some parts of the world where there is insufficient food and nutrients to feed the entire population. Malnutrition has been defined as the condition where nutrients are present in unbalanced quantities in the human body, leading to adverse health effect (5). Hidden hunger, also known as micronutrient deficiency is an undernutrition condition that arises from consuming foods rich in calories but are limited in minerals and vitamins (5, 6). Hidden hunger is very common in developing countries, particularly Sub-Sahara Africa and Southern Asia due to over-consumption of staple foods, changes in dietary patterns and inability to access adequate foods due to poor political and economic status (5, 7). Deficiencies in micronutrients such as Fe, iodine (I_2_), vitamin D and E have also been reported in the USA, Canada, and European countries (8, 9). The effects of malnutrition and hidden hunger have been drastic. Both have been devastating, particularly in infants, with an estimated 1.1 million out of 3.1 million infant deaths attributed to micronutrient deficiencies annually (5, 6). This has influenced various governments and stakeholders globally to devise strategies to tackle these challenges. These efforts include several initiatives such as the New Alliance for Food Security and Nutrition, The Scaling Up Nutrition Movement and The Harvest Plus Challenge Program (10–12).

Although dietary diversification and food supplementation have been used as intervention strategies, food fortification particularly biofortification has been projected to be the most sustainable intervention (13–15). Food fortification deals with the addition of selected nutrients to foods, naturally present in the foods or exogenous with the purpose of increasing the nutritional value of the food to help consumers reach the Recommended Dietary Allowances (RDAs) for those nutrients (6, 16). Biofortification, a form of food fortification involves the increase in the quantities and bioaccessibility of nutrients in food crops during their growth (16). Biofortification addresses the nutritional needs of both urban and rural populations and could be implemented at low costs. Multiple nutrients can be biofortified into foods without influencing the prices of foods (3). It does not require robust facilities; hence it is relatively easy to implement and does not depend on the compliance of the consumer (16). The Harvest Plus Program was initiated in 2003 to target Asia and African countries to ensure the nutrient availability and accessibility of high-quality biofortified varieties of staples. Common examples of biofortified crops through this program include orange-fleshed sweet potato (OFSP), golden rice, yellow, and orange maize biofortified with vitamin A, wheat and rice biofortified with Zn, Fe, and beans biofortified with Fe (15, 16). PBFs form a greater percentage of diets in certain populations hence their biofortification is relevant in combating malnutrition and hidden hunger.

Agronomic biofortification, plant breeding and transgenic approaches are some currently used strategies (1, 14). Agronomic biofortification involves the application of mineral fertilizers to soil or directly on crops to increase the content and bioaccessibility of specific nutrients in food crops (17). Biofortification through plant breeding aims at improving the concentration and bioaccessibility of minerals in crops by utilizing the genetic differences between crops of similar species (18, 19). There may be limited genetic variations among crops, making it impossible to biofortify certain crops *via* plant breeding. Alternatively, transgenic approach entails identifying and characterizing suitable genes which could be introduced into such crops to translate into desirable nutritional qualities (14). Crops such as cereals, legumes, oilseeds, vegetables, and fruits have been biofortified through these three strategies, with cereals being prominent. The limited genetic variability in oilseeds lends itself to the transgenic approach (14).

Recently, omics technology has been introduced to improve the efficiency of transgenic approach as a strategy of biofortification (20, 21). It involves identifying suitable genes through genomics/transcriptomics; overexpressing the desired gene through various transformation methods; studying the function of proteins in nutrient synthesis, uptake and transport pathways through proteomics; assessing metabolic pathways that control the biosynthesis of natural metabolites through metabolomics; and assessing the response of minerals to environmental and genetic factors through ionomics (21, 22).

**FIGURE 1:**
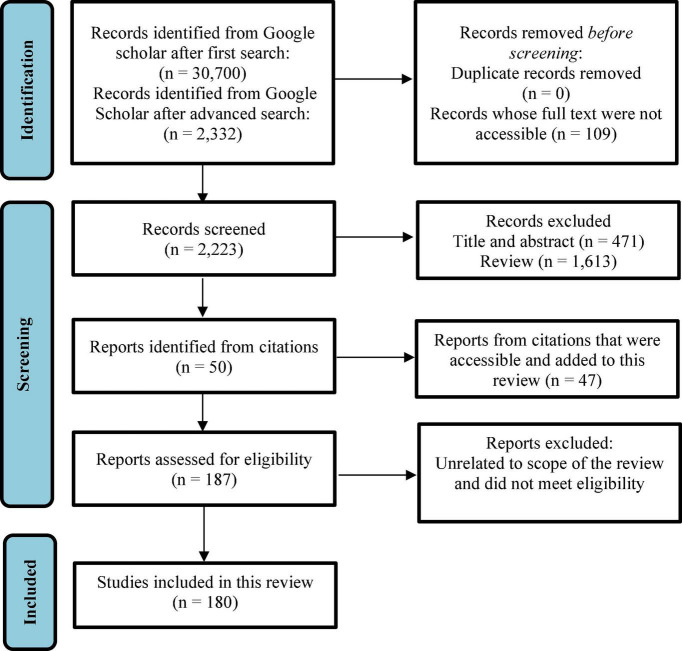
Flow diagram of information search for the systematic review (23).

This review sought to comprehensively present current interventions and initiatives adopted in combating malnutrition and hidden hunger in both developing and developed countries including the three main biofortification strategies and omics technology. The search and selection of studies, eligibility criteria, data extraction and risk of bias used in this systematic review is summarized in Figure 1 (23). The main findings include the important roles played by micronutrients in human health. However, in most developing countries, these micronutrients are limited in most diets. Among the interventions which have been utilized in improving micronutrient contents of foods, modern biofortification strategies such breeding and transgenic approaches have been the most effective in crops such as maize, rice, wheat, cassava and OFSP. The introduction of omics and gene editing techniques has enhanced the transgenic approach. Most research studies on biofortification of specific crops used in this review reported positive effects of biofortification on increasing micronutrient contents, bioaccessibility, and bioavailability. Although, political and economic influence, consumer acceptability, and regulations have proven to be the limiting factors of biofortification, it is still considered the best and sustainable strategy in tackling micronutrient deficiencies in plant-based foods (PBFs).

## Importance and micronutrient requirements through the life cycle

Food and nutrients are the chemical fuel utilized by the human body for metabolic activities, growth and development (1). Macronutrients including carbohydrates, proteins and lipids contribute greatly as the main sources of energy to the human body (24, 25). In contrast, micronutrients mainly minerals and vitamins, are required in smaller quantities to ensure proper functioning of the body (24, 26, 27). At each stage of human development, these nutrients are required in acceptable quantities daily (26) to ensure maximum growth and development. Minerals such as iron (Fe), zinc (Zn), iodine (I_2_), calcium (Ca), and phosphorus (P) are required for growth in children, adolescents and adults (24). Iron is important for the synthesis of hemoglobin and the functioning of the red blood cells in oxygen transport and energy production (28). Additionally, it aids in brain development, cellular metabolism and enzymatic functions (26, 29, 30). When children become deficient in Fe, the level of their physical activities, immune responses, brain and perception control reduces (24). Adolescents have increased Fe requirements due to its relevance in muscle development and formation of new red blood cells especially in adolescent girls who experience menstruation (24). Also, pregnant and lactating female adults have increased Fe requirements due to the need to deposit Fe for infants (26, 28). The recommended Fe intake for children, adolescents and adults are 7–11, 13, and 18 mg/day, respectively (24, 26). Zinc is essential for children due to its role in regulating growth hormones, promoting cellular immunity and gastrointestinal systems (24, 28). Growth occurs rapidly in adolescents, hence have higher Zn requirements (24). It is also essential for enzymatic activities in the body of adolescents and adults (26). Common symptoms of Zn deficiency include growth retardation, loss of appetite and weakened immune system (24). The recommended intake of Zn for children, adolescents and adults are 2.9–4.3 mg/day with upper intake levels of 7, 11, and 11 mg/day, respectively (24, 26, 31). Iodine plays an important role in the functioning of the thyroid gland in children, adolescents and adults (26, 32). Its deficiency is characterized by goiter and hypothyroidism especially in children (24). Children require 90 μg/day of I_2_ whilst adolescents and adults require 0.15 mg/day (24, 26). Due to the physical activities of children, it is important to consume adequate amounts of Ca and P for bone development (24). Adolescents have the highest Ca requirements due to rapid bone growth and increased physical activity within this phase of human development (24). Adults require adequate levels of Ca for proper functioning of the muscles and digestive system, and P for energy production (26). Children suffering from Ca and P deficiencies show symptoms of weak bones and rickets (26). The recommended intake of Ca for children, adolescents and adults are 280–450, 1150, and 1200 mg/day, respectively (24, 26), while for P, 460–500, 1250, and 700 mg/day are required for children, adolescents and adults, respectively P (24, 26).

Other minerals such as magnesium (Mg), selenium (Se), potassium (K), and silicon (Si) also contribute to human growth especially in adults. Mg is responsible for muscle contraction, enzyme functioning, synthesis of nuclear materials and transport of ions across membranes (26, 33). The recommended intake of Mg for children and adults are 170 and 320–420 mg/day, respectively (31, 33). Se promotes the activity of antioxidant enzymes and biological systems in the body (34). However, Se is required in extremely minute quantities in the body. The recommended intake of Se for adults is 0.055 mg/day (26). K acts as electrolyte for the body and regulates ATP for energy production (26). The recommended intake of K for adults is 4700 mg/day. The biochemical functions of Si have not been clearly defined and not considered as an essential mineral for growth (35). However, recent studies show that Si may be important for the functioning of bone and connective tissues (35). The recommended intake of Si has been set at 25 mg/day for adults (35).

Vitamin A plays an important role in maintaining vision, regulating cell and tissue growth, and improving the immune system (26, 36). The recommended intake of vitamin A for children and adults are 300–600 and 700–900 mg of RAE/day (retinol activity equivalent), respectively (36). Vitamin D is one of the essential vitamins for children due to its role in Ca absorption for bone tissue development (24). Additionally, it provides anti-inflammatory and anti-microbial properties in adults (27, 37). The recommended intake of vitamin D for both children and adults is 15 μg/day (24, 26). Vitamin K promotes coagulation and prevents excessive bleeding in children (38, 39). Additionally, it aids in bone and muscle metabolism in adults (40, 41). The recommended intake of Vitamin K for children and adults are 12 and 120 μg/day, respectively (24, 26). Vitamin C (ascorbic acid) plays a major role in enzymatic and non-enzymatic reactions, and acts as an antioxidant to improve the body’s defensive mechanism against diseases in both children and adults (27, 42). Deficiency in Vitamin C can be seen through symptoms such as tiredness, weight loss and scurvy (43). The recommended intake of vitamin C for children and adults are 20 and 90 mg/day, respectively (24, 26). The human body cannot synthesize folate therefore, it is essential to consume folate-rich foods (44). Folate is one of the essential nutrients for all stages of human development. It promotes rapid growth through cell divisions and DNA replication (24, 45). During pregnancy, folate deficiency results in neural tube defects in infants (46), thus the highest amount is required during pregnancy. The recommended intake of folates for children are 120 and 400 μg/day each for adolescents and adults (24, 26). Vitamin B12 is essential for the synthesis of DNA and hemoglobin and may be deficient in adolescents and adults who consume exclusive plant-based diets (47, 48). The recommended intake of vitamin B12 for children, adolescents and adults are 1.5, 3.5, and 2.4 μg/day, respectively (24, 26).

## Malnutrition and hidden hunger

Malnutrition is the generic term for the condition where food nutrients are present in excess (overnutrition) or limited quantities (undernutrition) in the body, leading to adverse health effect (5). As suggested by its name, hidden hunger denotes an undernutrition condition that arises from consuming foods rich in calories but is limited in minerals and vitamins with a sense of satisfaction to the consumer. Hidden hunger describes micronutrient deficiency unbeknownst to the consumer (6, 7).

Several factors synergistically contribute to malnutrition and hidden hunger, especially in developing countries (5, 7). These factors include climate change which affects food production, spikes in food prices which affect the accessibility of foods to the low-income populace, economic status of the populace and diseases such as cancer and chronic renal failure, which increase the risk of malnutrition, and hidden hunger in affected patients. Climate change has already affected food production by affecting changes in weather patterns, drought, and flooding. For example, in 2020, a locust swarm devastated wheat crops, leading to food insecurity, particularly in the most disadvantaged communities in parts of South Asia and Sub-Saharan Africa (16). Recently, most of the countries in Southern Africa including Angola, Botswana, South Africa, and Zimbabwe, experienced severe drought which started in 2016 but intensified in 2019 and 2020. All these countries depend on rain-fed agriculture as means of providing food to feed their populations (49). A study in South Africa showed a 23% decrease in field crops agriculture and agricultural-based industrial output reducing by 3.5% below the average as a result of severe drought. This negatively affected the country’s GDP and economy. The reduction in agricultural output had adverse effects on food security in the country (50). Similarly, impact assessment by FAO on the Sudan flooding showed that the flooding adversely affected 597,689 farms and pastoral households that were engaged in the rain-fed agriculture, causing damage to about 1,044,942 tonnes of crops. This has had limiting effects on food security in the country since about 55% of the affected populations depended on agriculture as source of income and food (51).

In both developing and developed countries, hunger, poverty, and malnutrition are interrelated challenges. Most people who suffer persistently from malnutrition are submerged in a vicious cycle; not being able to get nourishing meals regularly and consequently not being able to live an active and healthy life, not receiving adequate healthcare, hence not being able to produce or buy essential nutritious food. There is a close and complex relationship between hunger, malnutrition, and poverty. This phenomenon is described as the poverty trap, in which the poor are hungry, and their hunger traps them in poverty (25). On the other hand, the current COVID-19 pandemic has brought to light the vulnerability of the food system too, which has resulted in disruptions in food production and distribution. The international border closures have also impacted the labor force, particularly seasonal migrant workers working in agricultural fields (16). Movement restrictions have also had a negative impact on the transportation of food to marketplaces and limited consumers’ access to those, particularly in situations where open marketplaces are the primary distribution channels. There has been an inevitable loss and waste of food due to the outbreak, with crops that could not be harvested or transported to the marketplaces sitting in the field to rot and milk being thrown away due to interrupted supply chains.

### Trends in malnutrition and hidden hunger in developed and developing countries

The 2nd Sustainable Development Goal is aimed at eradicating extreme hunger and malnutrition whilst promoting food security and sustainable agriculture (2, 3, 16). This continues to be a big challenge globally but predominate in developing countries, particularly Sub-Saharan Africa, and Southern Asia. The Global Hunger Index (GHI) was updated in 2017 based on deficiencies in both calories and micronutrients using 119 countries as case studies (52). The report showed that one country falls in the extremely alarming range, 7 countries fall within alarming range, 44 countries within the serious range, 24 countries within the moderate range, and 43 countries within the low range on the GHI severity scale. In addition, the remaining 13 countries did not have sufficient data for the studies. However, 9 of the 13 countries with no sufficient data showed significant levels of hunger. The countries who suffered greatly from all forms of hunger were Southern Asia and Sub Saharan African countries with Yemen, Central African Republic, Chad, Liberia, and Madagascar being among the predominant countries (52). The prevalence of hidden hunger in developing countries has been attributed to their inability to access adequate diets due to their low income (5), over-reliance on staple foods that have limited micronutrient contents and changes in the type of diets from traditional less-processed foods to highly processed foods (6) and to the inferior quality of foods consumed, poor economic status, and political systems (52).

**FIGURE 2:**
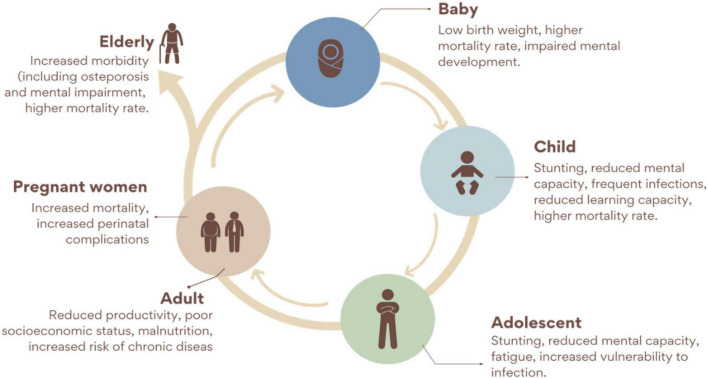
Consequences of micronutrient deficiency through the life cycle [Modified from (56)].

Although hidden hunger is not prevalent in developed countries compared to developing countries, Fe and Zn deficiencies can be found in most developed countries (6). Surveys in the USA, Canada, Great Britain, and other developed countries showed that the RDA for micronutrients such as Fe, iodine, vitamin D, and E were not met by a certain percentage of the population (8, 9, 25). This was attributed to changes in the dietary lifestyle in those developed countries, which have had substantial effects on the consumption of balanced diets and increased the occurrence of hidden hunger in those countries. The major micronutrient deficiencies in most developing countries include Fe, I_2_, Ca, Zn, folic acid, and Vitamin A deficiencies (5, 53). These deficiencies have had adverse effects on more than an average percentage of the world’s population (5). An estimated two billion people have suffered from hidden hunger globally (7, 54, 55). Among all the other micronutrient deficiencies, Fe deficiency is said to have affected a greater percentage of the human population worldwide (5, 9). Iron and other micronutrient deficiencies have direct relationships with mortality rates in infants and reproductive women (9). These micronutrient deficiencies are highly fatal, particularly in infants, with an estimated 1.1 million out of 3.1 million infant deaths attributed to micronutrient deficiencies annually (6). Aside infant and maternal deaths, malnutrition and hidden hunger also hinder growth, development, cognitive ability and physical working capacity (5). Furthermore, hidden hunger reduces productivity in the affected population which in turn affect the economy (5, 6) (Figure 2). These adverse effects of malnutrition and hidden hunger comprehensively explain why there is the need to find suitable ways of addressing them.

Undernutrition persists throughout life. A baby born with low birth weight grows up as a stunted adolescent and later becomes an under nutrient adult increasing the risk of chronic diseases, besides other adverse effects, including reduction of productivity. In parallel, malnourished women during pregnancy gain less weight, which increases their risk of delivering small infants. It has been demonstrated that this cycle extends over more than two generations through changes in DNA. Pregnant women, children, and adolescents are often the most cited as affected by hidden hunger; however, it adversely impacts people of all ages and stages (56).

Additionally, in some countries in Latin America and the Caribbean, there is a lack of data related to malnutrition (57). Many countries are usually left out from being included in the analysis, either because research teams are unwilling to participate or because there is no actual data to analyze. Moreover, anemia remains a public health issue among children under 6 years old and women in most countries for which data are available (58). Finally, other studies indicated that there is a high prevalence of Zn deficiency in children less than 6 years of age and girls and women from 12 to 49 years of age. High rates of both estimated Zn dietary inadequacy and stunting were also reported in most Latin American and Caribbean countries (57–59).

It is important to add that to successfully combat hidden hunger through biofortification, even after the development of biofortified varieties, it will be essential to address various socio-political and economic challenges to promote their cultivation and finally their consumption by customers. For future actions, an integrated approach is required, not only politicians and citizens need to be included but there is also a need to involve farmers, food product developers, dietitians, and educators. These stakeholders can impact population eating habits and contribute to increase the consumption of the target PBFs (60).

## Interventions to alleviate malnutrition and hidden hunger

The adverse effect of malnutrition and hidden hunger has been a global concern for several years forcing several countries to develop interventions. The 2008 Copenhagen Consensus, highlighted that governments and other agencies should prioritize the provision of micronutrients to their populace to serve as one of the best underlying factors for development (61). This has influenced several global initiatives including the New Alliance for Food Security and Nutrition formed in 2012 to help promote agricultural growth in Sub-Sahara Africa (10) and the Scaling Up Nutrition Movement sought to fight malnutrition around the globe (12). Several countries have added laws regarding nutrition and health to their constitutions (61, 62). Generally, there are four interventions that have been adopted in curbing malnutrition and hidden hunger. These include dietary diversification, food supplementation, food fortification and biofortification (1, 63).

### Dietary diversification

Micronutrients such as Fe, Zn, I_2_, and vitamins are limited in most staples but can be found in certain types of foods (5, 64). Therefore, consuming a narrow range of foods and more staples with insufficient quantities of micronutrient-rich foods may result in hidden hunger. This makes dietary diversification a vital strategy that can be used in curbing malnutrition and hidden hunger (65). Dietary diversification or modification pertains the consumption of different varieties of foods with sufficient quantities of macro and micronutrients that synergistically contribute to meeting the RDA over a specific period (6, 11, 16).

Dietary diversification helps alleviate all forms of deficiencies, aids in boosting the immune system, and is culturally acceptable, and sustainable as compared to other interventions used in alleviating malnutrition and hidden hunger (1, 66). The major disadvantages of dietary diversification as a strategy of alleviating malnutrition and hidden hunger include the obligation of nutrition education, change in dietary patterns, the need for accurate food data and presence of anti-nutrients in foods consumed, which impact nutrient absorption (6). Another disadvantage of dietary diversification is the need of financial commitment involved in purchasing and producing high-quality varieties of foods (5, 67, 68). Therefore, dietary diversification is very difficult to implement in developing countries; hence other interventions such as food supplementation, food fortification and biofortification are preferred in developing countries (5, 6).

### Food supplementation

Food supplementation involves the intake of additional nutrients in the form of capsules, syrups or tablets to add to the nutrients that are obtained from the consumption of foods to meet the RDA requirement (6, 69). Food supplements include vitamins, amino acids and proteins, essential fatty acids, mineral, fiber, and calorie supplements (69, 70). In most developing countries, vitamin supplements are most commonly used in combating hidden hunger whilst mineral supplements such as Zn, Fe, and amino acid supplements are less common (5, 6, 60). Food supplements are usually targeted to small populations with acute nutrient deficiencies in developing countries. Food supplementation is also a short-term, direct and controllable strategy, and can be tailored to meet the specific needs of a targeted population. It yields positive results rapidly and is cost-effective as compared to other interventions such as dietary diversification (6, 69). A population-based study in Ceará, Brazil was conducted to assess the association between vit. A supplementation and child development using 1,232 children between the age of 0 and 35 months in 8,000 households (71). It was reported that children supplemented with vitamin A showed over 40% lower probability each for delay in development of cognitive and motor abilities, as compared to children without vitamin A supplement. One of the key messages of the study was that vitamin A supplementation had positive effects on child development; indicating food supplementation can be used to improve the nutritional status of a population with micronutrient deficiencies.

Zinc supplementation at infancy increases specific growth outcomes, especially after age 2 (16). Nevertheless, identifying those at risk of Zn deficiency is challenging due to the lack of a reliable diagnostic tool. Another challenge can be reaching rural population as it needs continuous distribution of the supplements (67). Other disadvantages of food supplementation include its reliance on the compliance of the targeted population, require well-defined structures to successfully implement in targeted populations and is highly unsustainable, especially in developing countries (1, 5, 16). Food supplementation may also cause toxicity, which has severe effects on the health of targeted populations (70).

### Food fortification

Food fortification involves the addition of selected nutrients to foods, whether they are naturally present in the foods or not with the purpose of increasing the nutritional value of the foods to help consumers reach the RDA for those nutrients (6, 72). Both food fortification and food enrichment contribute to increasing the nutritional value of foods, but there is a major difference between them (73). Food enrichment only involves replacing the nutrients lost during the processing of the food, whilst food fortification considers restoring lost nutrients and adding to nutrients that are already present in the food in insufficient amounts. There are several forms of food fortification; voluntary fortification, mass fortification, mandatory fortification, and target fortification (3). Voluntary fortification occurs when food processing companies optionally add nutrients to processed foods as it not mandated by the government (3). A typical example of voluntary fortification is the addition of nutrients such as Fe and vitamin A to breakfast cereals and wheat flours as seen in countries such as Gambia, Qatar and United Arab Emirates (3, 61). Mass fortification involves the addition of selected nutrients to commonly consumed foods of a specific population with the aim of preventing specific nutrient deficiencies (72, 74). An example of mass fortification is the fortification of rice which is usually consumed by greater percentage of the population of Asian countries like China (72, 74, 75). As suggested by its name, mandatory fortification deals with the addition of nutrients to foods as demanded by the laws and regulations of the government (3, 74). It is the most common among all forms of fortification. Most governmental regulations demand the addition of iodine to salts, Fe and folic acid to wheat flour, and vit. A to edible oils (3, 61). Target fortification has been described as a form of fortification designed specifically for a particular group within a targeted population to reduce a particular nutrient deficiency (74). An example of target fortification is the addition of nutrients such as Fe to infant formulas (74, 75).

Food fortification has become relevant in both developing and developed countries due to the changes in dietary patterns with increases in the consumption of processed foods (3, 6). Extensive food processing and storage conditions tend to reduce nutrients such as water-soluble vitamins and minerals of foods (76). Food fortification acts as the medium through which these lost nutrients are restored after processing whilst complementing insufficient nutrients. Common examples of fortified processed foods include iodized salts, Fe and folic acid-fortified wheat, vitamin D and calcium-fortified milk and, vitamin A-fortified rice and edible oil (3, 6, 61). In some contexts, implementation of food fortification is limited due lack of well-structured processing and distribution networks (5, 16, 60). Food fortification also tends to favor urban areas rather than rural regions, where there are often communities with higher socioeconomic status, combined with higher levels of health education (16, 67).

Food fortification is measurable, sustainable in developed countries, and implemented at low costs (6, 61). Fortifying foods with nutrients such as Fe, I_2_ and vitamins in developing countries has greatly reduced the prevalence of diseases associated with nutrient deficiencies (77). However, in developing countries, the higher prices of fortified foods makes it less appealing to consumers (67, 78). Thus, in developing countries, biofortification is considered as a complementary intervention in alleviating malnutrition and hidden hunger.

#### Biofortification

Biofortification seeks to increase the quantities and bioaccessibility of nutrients in food crops during their growth (65, 79). It focuses on producing crops with high levels of micronutrients in addition to agronomic traits such as high yield and disease resistance (67, 80). Biofortification differs from food fortification in that the former involves the addition of nutrients to food crops prior to harvesting whilst the latter adds nutrients to foods during post-harvest processing (3, 81). Food fortification repeatedly adds nutrients to foods whilst biofortification of varieties of food crops occurs once (74, 81).

Biofortification has been projected to be the most sustainable solution to malnutrition and hidden hunger (15). At present, Harvest Plus, the Biocassava project, and the National Agricultural Research Organization (NARO) are the major projects initiated for nutritional security *via* the development of biofortified varieties (55). The initiation of The Harvest Plus Program in 2003, aimed to improve the quality (nutritional value) of food crops through biofortification (11). The Harvest Plus Program targeted Asia and African countries to ensure the availability and accessibility of high-quality biofortified varieties of staples and the bioavailability of nutrients after consumption (11, 15). Interventions such as food supplementation and industrial food fortification usually benefit the people of developed and industrialized countries with little to no impact in most developing countries. On the other hand, biofortification targets the developing and rural world and extends greatly to the developed world as well (5, 6). To fully implement biofortification, there is a need to assess the bioavailability of the nutrients, set targeted nutrient levels, assess the nutritional requirement of the targeted population, and enhance the absorption and retention levels of nutrients when subjected to processing and storage conditions (15, 82).

In 2017, a total of 33 million people across Africa, Asia, Latin America, and the Caribbean consumed biofortified crops (83). Common examples of biofortification include OFSP, golden rice, yellow and orange maize biofortified with vitamin A, Zn and Fe biofortified-rice and wheat, and beans (15, 16). Biofortification programs implemented in most countries have yielded positive results. In Nigeria, a 6-month study involving two groups of pre-school children aged 3–5 years was conducted. One group was fed with foods prepared using biofortified (yellow) cassava while the other group was fed with white cassava. The finding showed that the status of vitamin A (determined using serum retinol and hemoglobin concentrations) of the group that consumed the biofortified cassava significantly improved relative to the group fed with white cassava (84). While in Rwanda, hemoglobin, serum ferritin, and body Fe levels increased among reproductive women after consuming beans biofortified with Fe (85) (Table 1). Biofortification has certain advantages over other interventions to alleviate malnutrition and hidden hunger. It addresses the nutritional needs of both urban and rural populations and could be implemented at low costs after the initial developmental stages (16). Multiple nutrients can be biofortified into foods without influencing the prices of foods (3). It is the most sustainable method among other interventions (6). Biofortification of nutrients into food crops has less impacts on their organoleptic properties (16, 25). The main disadvantage associated with biofortification is its inability to rapidly improve the nutritional status of populations who are highly deficient in nutrients (6).

**TABLE 1:** Adapted biofortified crops (16).

Biofortified crop	Target micronutrient	Countries where crop has been tested	References
Orange sweet potato	Vitamin A	Uganda; Zambia	(86, 87)
Beans	Iron	Uganda; Zimbabwe; Rwanda	(85, 88)
Cassava	Vitamin A	Nigeria; Democratic Republic of Congo; Kenya	(84, 89)
Maize	Vitamin A	Nigeria; Democratic Republic of Congo; Zambia; Zimbabwe	(90)
Pearl millet	Iron	India	(88, 91)
Wheat	Zinc	India; Pakistan	(92, 93)
Rice	Zinc	Bangladesh	(94)

## Biofortification of plant-based foods

Global production, consumption, and sales of PBFs have significantly increased (95). Additionally, vegetables can contribute to combating undernutrition, poverty, and hunger, since they can be locally cultivated and consumed (60). Many consumers opt for exclusive PBFs due to the established relationships with health improvement, reduction in environmental impacts, and promoting food security (95, 96). PBFs have also been shown to provide nutritional benefits, specifically increased fiber, vitamin K and C, folate, magnesium, beta-carotene, and potassium consumption (97). Additionally, Ca, I_2_, and Se present in vegetable-rich diet, are beneficial for optimal bone strength, blood pressure, hormone production, heart, and mental health (60). However, it is important that consumers of exclusive plant-based diets select and combine PBFs to help prevent the risk of micronutrient deficiencies (95); particularly vitamin B12 (needed for neurological and cognitive health), which is mainly animal-derived nutrient, unless supplemented or provided in B12-fortified products (97). PBFs also contain high levels of anti-nutritional factors such as phytates and tannins known to reduce the bioavailability of minerals by preventing their absorption in the intestine (98). Additionally, processes like polishing, milling, and pearling of cereals can reduce their nutritional value (99). Biofortification of PBFs presents a way to reach populations where supplementation and conventional fortification activities may be challenging (100) and may serve as an essential step in preventing nutrient deficiencies, especially among consumers of exclusive PBFs.

**FIGURE 3:**
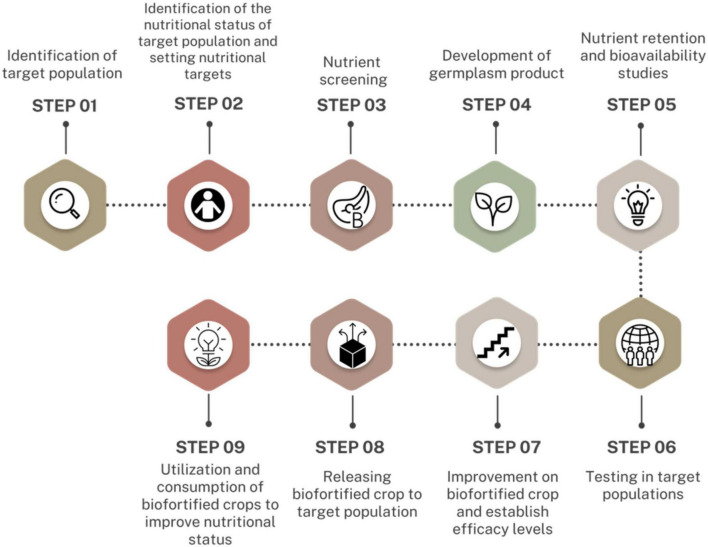
Harvest plus impact pathway–Plant-based biofortification steps [Modified from (101)].

Biofortification of PBFs involves increasing the levels of nutrients and their bioavailability. This is dependent on enhancing the bioaccessibility of the nutrients in the soil, uptake and transportation of the nutrients through the plant tissues, and their accumulation in non-toxic quantities in edible parts of the plants (22, 80). Biofortification of PBFs addresses two main challenges; the inability of the plants to synthesize certain nutrients and the uneven distribution of nutrients in different parts of the plant (22, 80). For example, the grains of rice are the consumed portion of the rice plant, however, pro-vitamin A synthesis and accumulation occurs in the leaves hence limited in quantities and bioaccessibility in the edible portion. Therefore, biofortifying the rice plant with pro-vitamin A may enhance its accumulation and bioaccessibility in the grains (55). The Harvest Plus Program has designed suitable steps involved in biofortification of PBFs. These steps (Figure 3) can be grouped into four categories; breeding, nutrition and food technology, impact and socioeconomics, and consumer response (11, 15, 101).

Although increasing concentration of nutrients is an important factor in PBFs biofortification, it is relevant to prevent over-accumulation of these nutrients due to its tendency to adversely affect the edible part of the crop and enhance the accumulation of undesirable metals, which could affect consumer’s health (22, 55). For example, Se play important roles and is required in extremely minute quantities in the human body with recommended level of Se intake for adults being 0.055 mg/day (26). Excessive intake of Se causes toxicity which is characterized by adverse health effects such as muscle soreness, intestinal complications, cardiovascular diseases and can extend to extreme cases of mortalities (102). The consumption of biofortified foods should help consumers meet their nutritional needs without any risk of toxicity.

Both bioavailability (fraction of nutrient that is stored or available for physiological functions) and bioaccessibility (fraction of the total nutrient that is potentially available for absorption) (80) are important in PB biofortified foods. In a study reported by (103) significant improvement in contents, bioaccessibility and bioavailability of Fe- and Zn-biofortified cowpea cultivars were shown. The values obtained for Fe bioaccessibility [18.5–32.3 mg/kg (2.5-fold higher)] and bioavailability [>3.0% (2.6-fold higher)] in Fe-biofortified cowpea seeds were found to be higher compared to common beans (7.3–15.4 mg/kg) and (1–2%), respectively. Zn bioaccessibility in the biofortified cowpea cultivars was also about 25% higher than in common beans, while Zn bioavailability in the biofortified cowpea was slightly higher compared to the common beans. In another study, increasing doses of selenate during wheat biofortification enhanced both Se content (0.18–0.45 mg/kg) and bioaccessibility (63.3–93.8%). However, increasing the selenate doses did not necessarily improve the bioavailability of Se in both apical applications. The% bioavailability, based on the% bioaccessibility decreased from 82.6 to 62.6% for apical application with increasing doses of selenate. For the basal application, it increased from 17.7 (0 g ha^–1^ dose) to 24.4% (5 g ha^–1^ dose) and later decreased to 18.0% (2 g ha^–1^ dose) (104).

Although biofortification might improve micronutrients bioaccessibility in food crops; dietary, physiological, human, and genetic factors can affect the bioavailability of these micronutrients in the human body when consumed (105–107). Due to the presence of antinutrients, biofortified crops showed limited improvement in the bioavailability of certain nutrients (108, 109). Antinutrients such as phytic acid/phytate, tannins, lectins, saponins, and oxalic acid can limit the absorption of certain essential nutrients (25, 110). These include phytic acid/phytate mostly found in PBFs such as wheat, rice, barley and beans (110). Phytate/phytic acid accumulate in the seed during ripening and forms complexes with Fe, Zn, Ca, Cu, and Zn and reduces the solubility and absorption of these minerals (111). It also binds with proteins to form complexes which decrease their solubility, limiting nutrient digestion, release and absorption (25, 112). To overcome this problem, phytase may be added to degrade phytic acid, reducing its ability to form complexes and, enhance micronutrient absorption (110). Ascorbic acid forms complexes with Fe^3+^ and reduces it to soluble Fe^2+^ which is more bioavailable (112). Tannins, a polyphenol is mostly found in legumes, berry fruits and cocoa beans thereby causing reduction in Fe bioavailability by forming tannin-Fe complexes (25, 110, 113). Lectins are common in legumes, cereals and fruits and can damage the cells of the gut epithelium limiting its efficiency in nutrient absorption (110, 113). Saponins are commonly found in crops such as legumes, tea leaves and oats, and have the potential to form complexes with sterols which affect the absorption of fat-soluble vitamins such as vitamin A and vitamin E (113). Oxalic acids form strong bonds with Ca, Mg, K, and Na forming soluble or insoluble oxalate salts, which prevent the absorption of these nutrients for metabolic activities (111, 114). Furthermore, the effects of these anti-nutrients are at sub-lethal levels. Pre-processing and processing conditions such as soaking, germination, cooking, extrusion, milling, and chemical treatments have been used to reduce the antinutrient contents in foods (108, 111, 113). Although these antinutrients negatively affect the absorption of essential nutrients, they have health-promoting properties which could be beneficial to the body (115). Phytate has been shown to have hypoglycemic, anti-inflammatory and anti-carcinogenic properties (25, 115). Polyphenols aid in removing free-radicals and limiting low density lipoproteins (110, 116). Lectins promote mitotic cell divisions and destruct cancer-affected cells (114). Saponins have great antimicrobial, cancer-prevention and cholesterol-reducing properties which reduce the risk of cancer and heart diseases (112, 114). Therefore, a balance of these antinutrients/bioactive compounds may offer beneficial effects (115).

The food environment from which biofortified foods are consumed can influence bioavailability of micronutrients. When micronutrients are entrapped in macronutrients matrix, their bioavailability may depend on the breakdown of the macronutrient (117). The presence of fats in the food environment promotes the absorption of fats soluble vitamins such as vitamin D, subsequently enhancing Ca absorption (106). Dietary fibers with increased solubility tend to bind with minerals and reduce their bioavailability (117). Age is one of the human factors that influence bioavailability of micronutrients; increasing age decreases the bioavailability of micronutrients (105). Micronutrient absorption may increase at certain periodic stages such as pregnancy, lactation and breastfeeding. Bioavailability of calcium increases at these stages in women to meet the nutritional requirements of the infant (106). Also, the health condition of consumers may have limiting effects on bioavailability of micronutrients. Conditions such as diabetes, obesity, celiac disease, hypochlorhydria, chronic pancreatitis, and parasite infections limit the absorption of micronutrients (106, 118). People from different ethnic groups may have variations in genes involved in micronutrient absorption, affecting their bioavailabilities (119).

Three biofortification strategies including agronomic intervention, conventional plant breeding and genetic engineering have been described for PBFs (1). These strategies have been applied to cereals, legumes, oilseeds, vegetables and fruits, with cereals having the largest number of biofortified varieties. Due to limited genetic variability in oilseeds, the transgenic approach is well-suited (14, 55).

## Strategies for biofortification of plant-based foods

### Agronomic biofortification

Agronomic biofortification involves the application of mineral fertilizers to soil or crops to increase the concentration and bioaccessibility of specific nutrients in the crops (17). Initially, agronomic practices were done to improve the health of crops and increase yield. However, the importance of nutrition has been highlighted over the years; hence agronomic practices have been expanded to improve the nutritional qualities of crops (5, 65, 120). Changes in climate conditions and rapid depletion of soil nutrients is an indication of the need to improve and expand agronomic practices to include improving the nutritional qualities of crops (121). Agronomic biofortification focuses on improving solubilization and mobilization of minerals (55, 120). The effectiveness of agronomic interventions depends on the soil composition, the solubility and mobility of minerals, the ability of crops to absorb minerals, and the accumulation of bioavailable minerals in non-toxic levels in the edible parts of the crops (25, 55, 80). Agronomic biofortification mainly covers minerals and not vitamins because vitamins are synthesized in the crops. Hence, agronomic biofortification cannot be used as a single strategy in eliminating micronutrient deficiencies and should complement other strategies for effective biofortification (1, 5, 55). The use of fertilizers for agronomic biofortification must be performed carefully as an improper application of fertilizer can have unanticipated, and sometimes severe, consequences on the environment and crops. In contrast, a balanced fertilization strategy is both economically more beneficial and environmentally more sustainable. Additionally, soil microorganisms play a crucial role in the soil ecosystem and are highly sensitive to fertilization. A deficient fertilization regime results in nutrient deficiency and subsequent modifications of the microbial community of the soil. Unbalanced fertilizations can have detrimental effects on soil biological health over the long-term (55, 122).

Mineral fertilizers are mostly applied to the soil or directly sprayed on the leaf of crops. The former is more common and applicable when nutrients are required in higher amounts. Foliar application is more economical and applicable when nutrient deficiency symptoms in crops are visible (25, 120), when mineral elements are not translocated and accumulated in adequate amounts in the edible parts of the crop (1, 120). Foliar application tends to be more effective than soil applications because unlike soil application, it increases micronutrient contents rather than just promoting yield (25, 120, 123). Foliar application is dependent on several factors including the type of fertilizer, characteristics of crops, time of application, and environmental conditions (115, 124). Agronomic biofortification of crops with minerals such as Fe and Zn require certain adjustments. Due to their low mobility, adding metal chelators to the fertilizer is essential (5). Foliar application of FeSO_4_ has proven effective for Fe biofortification (125). For I_2_, potassium iodate has been effective as seen in countries like China (5, 126, 127). Inorganic fertilizers such as ZnSO_4_, ZnO, and Zn-oxy-sulfate are suitable for Zn agronomic biofortification. Just like Fe, foliar application of Zn chelators such as ZnEDTA is highly effective (128–130). Se is agronomically fortified as selenate which is converted into organic selenomethionine in the crop. Both foliar and soil applications are suitable for Se biofortification, but dependent on soil type and timing of the application (131). However, foliar applications are costly and could easily be rinsed off by raining water (120, 131). The characteristics of the leaf play an important role in absorbing nutrients during foliar applications. Nutrients from foliar application penetrate the cuticle to leaf cells and are transported to other parts through the plasmodesmata. The age, structure and permeability of the leaf affect nutrients absorption (124). Foliar application is mostly effective during the flowering and early milk phases than booting and elongation phases of the developmental stages of crops. The flowering and early milk stages are among the earliest phases where absorption of nutrients for fruit formation begins, hence, foliar application of nutrients at this stage would contribute greatly to increasing the micronutrient contents of the fruits (123, 132). This was experienced during Zn agronomic biofortification of wheat using foliar application, which was attributed to enhanced phloem mobility and active photo-assimilate allocation to reproductive silk organs that enhanced remobilization of nutrients (115, 123, 132). Also, environmental conditions such as time of the day, humidity, temperature, and wind speed affect the efficiency of foliar applications (124). Warm and moist conditions in the early morning and late evening promote permeability of nutrients whilst low relative humidity and high temperature evaporate water from sprayed solution, leading to concentration of minerals on surfaces which reduces mineral permeability (124). Other strategies that are used for agronomic biofortification include coating and priming of seed with mineral fertilizers. These strategies aid in promoting crop yield and development but have minimal effects on the nutritional qualities of crops (55, 120).

Agronomic biofortification has been used effectively in several countries to combat micronutrient deficiencies and promote agricultural productivity. The effect of agronomic biofortification of selected underutilized vegetables in Ghana has been assessed (17). Increasing application rate of K fertilizer increased fruits and vegetables weight. Also, the application rate of K fertilizer and the type of K fertilizer synergistically affect K concentration in the fruit. The highest fruit K concentration was reported to be 2316 mg K kg^–1^ DW and this was a 140% increase relative to the control (no K fertilizer application). In another study that assessed the influence of irrigation and fertilizer application on β-carotene yield and productivity of OFSP in South Africa (133), the total storage root yield increased by 2–3 folds and β-carotene content increased from 133.7 to 151.0–153.1 μg/g when 50–100% fertilizer was applied, compared to no fertilizer application.

Agronomic biofortification is simple and yields results rapidly in the short term (5, 131). However, mineral fertilizers used in agronomic biofortification is costly which increases the prices of biofortified crops, making them inaccessible to poorer populations (129). Also, agronomic biofortification is highly dependent on farmers. Application of mineral fertilizers is a regular activity hence may be omitted by farmers if they do not gain profits from the process (25, 80, 120). Application of mineral fertilizers repeatedly may also cause accumulation, leading to toxicity (1, 120). In addition, increasing demand for mined minerals such as Se may cause exhaustion and negative impact on the environment (80, 120).

### Plant breeding

Plant breeding involves producing genetically different or new varieties of crops with improvements in essential micronutrients (55, 134, 135). Biofortification through plant breeding aims at improving the concentration and bioaccessibility of minerals in crops by utilizing the genetic differences between crops of similar species (19, 55). Plant breeding initially focused on promoting yield and improving agronomic traits of crops however, recent plant breeding techniques have been geared toward promoting both the nutritional quality and agronomic traits (54, 135). Plant breeding techniques should focus on introducing genotypes that would enhance the uptake, transport and redistribution of minerals to improve the efficiency of biofortification (136). In order to achieve this goal, there is a need to enhance mineral mobility in the phloem vessels responsible for redistributing and remobilizing these minerals (136). The translocation and redistribution of Zn from the shoot to fruits or edible portions of crops has been a challenge due to the low mobility of Zn in phloem vessels, leading to lower Zn concentrations in the edible portions as compared to the leaves or the root system (60, 136). Plants have been bred using three main techniques–conventional, molecular and mutation breeding (25, 55).

Conventional breeding is the most common and accepted form of plant breeding for biofortification (14, 55). Conventional breeding enhances improvement in the nutritional qualities of crops without compromising other agronomic traits (54, 55, 135). Biofortification through conventional breeding involves crossing crops with genotypic characteristics of high nutrient density and other agronomic traits to produce new varieties with desirable nutrient and agronomic traits (14). It requires identifying the biodiverse varieties of crops, assessing traits and amounts of target nutrients in these varieties, and determining the effects of growing conditions on the stability of these traits (137). Currently, about 299 varieties of biofortified cops have been released in over 30 countries *via* conventional breeding (135). A typical crop biofortified through conventional breeding is OFSP which has been biofortified with pro-vitamin A and with increased yield traits (19, 135, 138). Quality Protein Maize (QPM) is also a product of conventional breeding (25, 55). Other recent examples of conventionally bred biofortified PBFs include biofortified wheat varieties, “Zincol” and “Akbar-2019” released in 2015 and 2019, with enhanced Fe and Zn contents, Fe-biofortified beans and, pro-vitamin A-biofortified cassava and maize (19, 134, 139).

Mutation breeding differs from conventional breeding such that, differences in genetic traits among crops are created by introducing mutations through chemical treatments or physical methods such as irradiation (25, 55). Mutation breeding has been recently adapted to biofortify resistant chickpea mutants like Pusa-408 (Ajay), Pusa-413 (Atul), Pusa-417 (Girnar), and Pusa-547, developed at I.A.R.I., India. Crop improvements *via* mutation in Pusa-547 include: thin testa, attractive bold seeds, better cooking quality and high yield performance (140). Unlike conventional breeding, differences in genetic traits among crops are created by introducing mutations through chemical treatments or physical methods such as irradiation (25, 55).

Biofortification through molecular breeding involves identification of the position of a gene responsible for improving the nutritional quality and closely linked markers to that specific gene. With the aid of the marker, the desirable traits can then be bred into the crop using conventional breeding (1, 55). Molecular breeding can be used to determine if a desirable trait is present or absent in a specific crop during developmental stages. Hence, it is more rapid as compared to other forms of plant breeding (25, 55). Molecular or marker-assisted breeding has been used to develop several varieties of maize with improved pro-vitamin A content which can provide 25–50% of the estimated average requirements for vitamin A for women and children (55, 141). These varieties have been released in countries such as Zambia, Nigeria and India. Also, it has been reported that several rice varieties have been bred to produce a variety with high Fe and Zn contents and improved agronomic traits (14).

Plant breeding is sustainable and less costly as compared to other biofortification strategies (1, 14), and financial investments occur only at the research and development stages. Also, unlike agronomic biofortification, plant breeding has little to no impacts on the environment (19). Consumers generally accept crops that are biofortified through conventional plant breeding and easy to obtain regulatory approval as compared to genetically modified (GM) foods (19). However, conventional breeding is labor intensive and takes longer time to develop varieties with both desirable traits such as nutrient densities and agronomic traits (19, 22). Also, there may be limited genetic variations among crops, making it impossible to biofortify these crops *via* plant breeding (14, 79, 134), and may not be successful for all nutrients. For instance, breeding varieties of rice with improved vitamin A content initially proved to be challenging, but recent advances in omics technologies have provided the opportunity to practically biofortify varieties of rice with pro-vitamin A (79). Also, crops such as banana that are propagated by vegetative means are not suitable for conventional breeding (79).

### Genetic engineering/transgenic approach

Plant breeding relies heavily on genetic variations among crops and when variation is limited, it hinders the opportunity of biofortification through plant breeding (135). Unlike plant breeding, genetic engineering is not limited to crops of related species. Genetic engineering has demonstrated to be a viable solution to this problem (14, 65) and has been shown to effectively biofortify crops such as banana and rice, which cannot be subjected to conventional plant breeding (79, 135, 142). Genetic engineering provides the platform for introducing nutrient or agronomic traits new to specific crop varieties by applying plant breeding and biotechnology principles (123, 143) and when employed in biofortification, it identifies and characterizes suitable genes which could be introduced into crops to translate into desirable nutritional qualities (14). It utilizes genes from vast array of species, including bacteria, fungi and other organisms. Certain microorganisms enhance the uptake of nutrients by plants. Genes from these microorganisms can be genetically engineered into crops to enhance nutrient absorption, transportation, and concentration (25, 143). Fluorescent pseudomonas is a bacterium that enhances plant Fe uptake. Plants growth-promoting rhizobacteria and mycorrhizal fungi enhance the absorption of minerals from the soils and promote plants growth. Genes from bacteria and *Aspergillus* species have been used to adjust the lysine and phytate contents of crops such as rice and wheat, respectively (25, 55).

Genome editing, also known as gene editing, corrects, introduces or deletes almost any DNA sequence in many different types of cells and organisms (144). Gene editing provides an opportunity to develop GMOs without the use of transgenes; in addressing regulatory challenges associated with transgenic crops (143, 145). Methods such as Mega-nucleases, Zinc-finger nucleases (ZFNs), transcription activator-like effector nucleases (TALENs), and Clustered regularly interspaced short palindromic repeats (CRISPR/Cas9) have been exploited in genome editing to produce β-carotene biofortified rice and zinc-rich wheat varieties (65, 146). Except for the CRISPR/Cas9 technique, these genome editing methods are complex, expensive, and labor-intensive (65). Although CRISPR/Cas9 is flexible, cost-effective, and precise, it can sometimes lead to undesired mutations when untargeted regions in the genome are involved in the editing process (65, 145). These off-targets can be overcome by the dimeric nuclease method, which is highly precise and specific (145). According to (147), low levels of knowledge about gene editing occur because information generated in scientific studies has not been communicated effectively to consumers (148).

Biofortification through transgenic approach has been greatly explored in most developed countries. The most notable example is golden rice which was developed by biofortifying rice with pro-vitamin A (1). This was done by expressing genes encoding phytoene synthase and carotene desaturase which are responsible for β-carotene pathway (121). In golden rice, the expression of these genes caused an increase in pro-vitamin A levels by 1.6 to 3.7 μg/g DW (121). The overexpression of *Arabidopsis thaliana* vacuolar Fe transporter VIT1 in cassava caused 37-fold increase in Fe contents in the storage roots (149). The overexpression of Zn transporters and expression of the gene responsible for phytase activity in barley enhances the levels and bioavailability of Zn (55, 79).

In order to improve the efficiency of the transgenic approach as a biofortification strategy, omics technology has been introduced (20, 21). Omics technology explains the interrelationship between genes, proteins, transcripts, metabolites, and nutrients (20–22). Specific genes control the uptake, transport, concentration, and bioavailability of nutrients by crops. Hence, genomics (omics technology of genes) is important since it presents the opportunity to study these specific genes and design suitable ways of improving and inducing them into crops (138, 150). Transcriptomics (omics technology of transcripts) aids in conducting full-spectrum analysis to identify a specific expressed gene (20, 151, 152). Proteomics (omics technology of proteins) helps to understand the role of proteins in nutrient synthesis, uptake and transport pathways (21, 22). Metabolomics (omics technology of metabolites) aids in assessing metabolic pathways that control the biosynthesis of natural metabolites (153, 154), while ionomics considers how minerals present in crops undergo changes in response to genetic and environmental factors (22). These omics technologies have been used in studies involving biofortification of lysine, Ca, Zn, Fe, and vitamin C in PBFs such as maize, finger millet, wheat and tomatoes, respectively (155). PBFs such as cauliflower, cassava, and banana have been biofortified by both transgenic and breeding approaches while barley, soybean, lettuce, canola, carrot, and mustard have been biofortified with transgenic and agronomic approaches (14). The transgenic approach has been shown to be sustainable and rapid when introducing desired traits into crops (25, 143). Table 2 summarizes a selection of biofortified crops developed by transgenesis.

**TABLE 2:** Some examples of biofortified crops produced by transgenesis.

Crop	Gene/Protein	Target and country	Status^1^	References
Soybean	Phytoene synthase c*rtB*	β-carotene	Released	(143, 156)
	FATB1-A and FAD2-1A	Reduced linoleic acid, Vistive Gold^®^ (United States)		(145)
Maize	*Aspergillus niger phy*A2	Phytate degradation; BVLA4 3010 (China)	Released	(157)
	*Corynebacterium glutamicum cordapA*	Lysine; Mavrea™ YieldGard (Japan and Mexico)		(145)
		Lysine; Mavrea™ Maize (LY038) (Australia, Colombia, Canada, Japan, Mexico, New Zealand, Taiwan, United States)		
	Ferritin and lactoferrin	Iron	Research	(65, 158)
Rice	*C1* and *R-S PAL, F3′H, ANS, CHS*, and *DFR*	Flavonoids	Research	(159, 160)
	*OsIRT1*	Zinc		(143, 161)
	Maize *psy*1, *Pantoea ananatis* bacterium *crtI*, and *E coli* strain K-12 *pmi*	Provitamin A rice line GR2E (Australia, New Zealand, Canada, United States)	Released	(145)
Cassava	Ferritin and *FEA1*	Iron	Released	(162)
	*Arabidopsis* ZAT and ZIP	Zinc		
	Phytoene synthase c*rtB* and DXS	β-carotene		
	*ASP1* and Zeolin	Protein		
Sweet potato	*Crtl, CrtB, CrtY, LCYe*	β-carotene (South Africa, Mozambique, Bangladesh, and other African countries)	Released	(65, 143, 163)
	*IbMYB1*	Antioxidants	Research	(164)
Banana	Phytoene synthase PSY2a	β-carotene	Research	(165)
Alfalfa	MtlFS1	Isoflavonoids	Research	(166)
Wheat	Ferritin *TaFer*	Iron	Released	(143, 167)
	Silencing *SBElla*	Amylose	Research	(168)
	*PSY, Crtl, CrtB*	Provitamin A, carotenoids		(169, 170)
Potato	nptII	Amylopectin component of starch; AM 04-10200 (United States)	Released	(171)
		Amflora™ (EH 92-527-1 (European Union)		
Tomato	HMT, S3H, and SAMT	Iodine	Research	(65, 172)
	GDP-l-galactose phosphorylase	Vitamin C		(65, 173)
Barley	AtZIP1	Iron, Zinc	Research	(65, 174)

^1^Status: Released means products are available in the marketplace; research means laboratory investigations are ongoing.

Biofortification through the transgenic approach has its limitations. The transgenic approach requires huge investments in financial, time and human resources at the research and developmental stages (1, 5, 25). Transgenic crops are not generally accepted due to concerns over GMOs (5, 22). Also, there are several regulations governing the production of transgenic crops (137). Interactions among genes introduced into crops during genetic engineering may reduce the efficacy of the biofortification process (14, 22). Agronomic biofortification, plant breeding and genetic engineering, including omics technology, are suitable strategies that could be used for plant-based biofortification to help reduce the occurrence of malnutrition and hidden hunger.

## Regulations, consumer acceptance, opportunities, and future prospects

The successful implementation of biofortification programs depends on the acceptance of biofortified crops by farmers and consumers (143). The acceptance of GM crops is different for customers and farmers. In general, consumers have expressed a lower level of acceptance for GM crops and foods, because they are skeptical about the risks and benefits associated with these products (175, 176). Many factors influence consumer attitudes, including information, trust, beliefs, perceptions of benefits and risks. Several concerns have been raised about the human health implications from gene flow and transfer, environmental impacts from possible development of resistant weeds and crops, impacts on conventional methods, artificial-like methods, toxicity, and allergenicity of GM crops (145, 170). It is because of this that genetically engineered plants and their products are often rejected based on unverified grounds. The overall inclination toward avoidance have been directed toward GM crops, even though several scientific reports have shown that GM crops are safe to consume (170, 175). Therefore, it may be necessary to create adequate informational programs which would highlight the importance of biofortified-genetically engineered plants and quell misconceptions about GM crops (143). In many parts of the world where biotech seeds are available, farmers are highly embracing and accepting biotech seeds because of the benefits they receive from GM crops (177).

Globally, GM crops are governed by different regulations (178). These regulations and legislations have huge effects on the commercialization and adoption of GM crops (179). Strict labeling rules of GM crops have been set by over 40 countries (170). The European Union introduced a very strict authorization system for GM crops and foods over a decade ago, as a precaution. All food derived from GM plants were required to be labeled based upon the process, even if no traces of the genetic modification could be detected in the purified end-product. Accordingly, recent trends in many European countries have created an environment that makes the cultivation of GM crops and foods extremely difficult. The US legislation is more lenient in that novel GM foods do not have to be labeled if they do not differ in composition from established non-GM foods. As with Europe, China has been requiring the labeling of foods derived from GM crops for more than a decade (177, 180). It was estimated that it can take about 13 years to develop and commercialize GM crops, and the average cost of acquiring authorization for commercialization of GM crops was about $35 million USD (145). Also, most agri-business companies patent newly developed GM crops, monopolizing the commercialization of the GM crops. Several debates have been raised about the motive for developing GM crops–for privatization and profit-making or for the purpose of promoting food security (170). Therefore, regulations and legislations governing GM crops should be adjusted, especially in developing countries, to be less rigorous, and cost and time-effective to promote the adoption of GM crops (112, 170).

In many countries, researchers and seed companies are predicting that new breeding techniques such as intragenesis and cisgenesis, which transfer only genetic information from the same species without transferring foreign genes, but could provide smoother routes to market for plants with improved traits, thereby avoiding the roadblocks presented by transgenic GMOs (145, 180). These newly developed breeding techniques do not fit within the traditional definition of GMOs, and a debate is taking place in many areas regarding how they should be regulated. In view of this regulatory uncertainty, it is possible for GM products to be distributed differently in the market and for customer acceptance to differ globally (175). What does the future hold for GM crops? Will there be a universal labeling system for GM crops? Should there be a change in regulations governing Intellectual property rights of GM crops? The adaptation of GM crops in the production of biodegradable polymers has been discussed. Does this indicate that GM crops have strong positive environmental impacts in the future (112, 170)? The possibility of using transgenic crops as means of providing vaccines and medications can be exploited in the future (170). Can GM crops be adapted to have other desirable traits aside agronomic and nutritional traits? These questions could be the focal points of future research in promoting the development and commercialization of GM crops.

## Conclusion

Malnutrition and hidden hunger are both present in developed and developing countries and have devastating effects globally. The recent implications of the global pandemic have shown that food systems need to be adapted to advance global changes that can limit deficiencies in our food supply. Furthermore, climate change projections predict higher inequality and poverty for developing countries and hence, the need to augment the nutritional content in PBFs. Biofortification is the most sustainable and cost-effective method for alleviating malnutrition. Biofortification of PBFs has been used to produce crops with adequate nutrient density and bioavailability and help to combat hidden hunger. Through plant breeding, transgenics, and mineral fertilizer applications, micronutrient malnutrition can potentially be tackled. It is important to add that to successfully combat hidden hunger through biofortification, even after the development of biofortified varieties, it will be essential to address various socio-political and economic challenges to promote their cultivation and finally their consumption by customers. For future actions, an integrated approach is required, where politicians, farmers, food product developers, genetic engineers, dietitians, and educators need to be included in the developing efforts. One of the biggest challenges of biofortification aside from the methods to strengthen the nutritional value of crops is the public acceptance. Especially for the transgenic techniques more education and marketing should be invested for the success of biofortified products in the market as only few cultivars are finally released for costumers. Globally, the specificity of biofortification techniques should tackle regional nutritional challenges and should be chosen based on the likelihood of acceptance of cultural difference in consumers. Overall, biofortification represents a promising group of techniques that can improve the global nutritional wellbeing and lead us closer to minimize hunger and malnutrition.

## Author contributions

AA conceptualized the manuscript, crafted the outline, led the manuscript writing, and formatted the final version of the manuscript. KO and SA wrote the initial draft. SA prepared the illustrations. AA and ME reviewed and edited the manuscript. All authors approved the submitted version of the manuscript.
